# Highly Dense TiO_2_ Nanorods as Potential Electrode Material for Electrochemical Detection of Multiple Heavy Metal Ions in Aqueous Medium

**DOI:** 10.3390/mi16030275

**Published:** 2025-02-27

**Authors:** Sadia Ameen

**Affiliations:** 1 Advanced Materials and Devices Laboratory, Department of Bio-Convergence Science, Jeongeup Campus, Jeonbuk National University, Jeongeup 56212, Republic of Korea; sadiaameen@jbnu.ac.kr; 2 Department of Bioactive Material Sciences, Jeonbuk National University, Jeonju 54896, Republic of Korea

**Keywords:** TiO_2_, heavy metal ions, nanorods, environmental remediation, electrochemical detection

## Abstract

This study describes the direct deposition of extremely dense TiO_2_ nanorods (NRs) on an ITO substrate for the improved detection of heavy metal ions (HMIs). A facile hydrothermal method was employed to synthesize TiO_2_ NRs on the ITO substrate at ~130 °C. Synthesized TiO_2_ NRs were analyzed for morphological, structural, and electrochemical properties. As an electrode material, TiO_2_ NRs were used for the simultaneous detection of three HMIs (i.e., Cr^3+^, Cu^2+^, and Hg^2+^), which showed a remarkably high sensitivity of ~92.2 µA.mM^−1^.cm^−2^ for the Cu^2+^ ion. Relatively low sensitivities of ~15.6 µA.mM^−1^.cm^−2^ and ~19.67 µA.mM^−1^.cm^−2^ were recorded for the Cr^3+^ and Hg^2+^ ions, respectively. The fabricated TiO_2_ NR-based HMI sensor showed an effective dynamic linear detection range with low LOD values of ~21.7 mM, 37 mM, and ~ 28.5 mM for Cr^3+^, Cu^2+^, and Hg^2+^, respectively. The TiO_2_ NR-based HMI sensor exhibited efficient charge transfer over the electrode toward the trace detection of Cr^3+^, Cu^2+^, and Hg^2+^. Moreover, the reliability of the TiO_2_ NR-based HMI sensor was assessed, which exhibited a promising stability of 30 days. The obtained results indicate that TiO_2_ NRs grown on an ITO substrate are a promising electrode material for detecting hazardous Cr^3+^, Cu^2+^, and Hg^2+^ and might eventually be commercialized in the near future.

## 1. Introduction

Modern industrialization is advancing so quickly that heavy metal contaminants are being released into the environment in large quantities, which has led to the serious contamination of food sources, soil, and water [1,2,3]. The biological chain can allow heavy metal ions (HMIs) to accumulate, which poses a serious risk to human health [4,5,6]. For example, it is well recognized that Cd^2+^ ions can lead to cancer and detrimental injury to the kidneys, liver, and bones [7,8]. Pb^2+^ ions adversely impact cardiovascular systems and are associated with the emergence of chronic hypertension and myocardial dysfunction [9,10]. Another HMI, the Hg^2+^ ion, causes irreversible harm to the reproductive and central neurological systems [11,12], whereas high levels of Cu^2+^ ions have been linked to negative consequences for human health, such as the possibility of irreversible DNA changes [13,14,15]. Other ions such as trivalent and hexavalent chromium (Cr) are frequently encountered in soil, seawater, and groundwater [16,17]; however, the excessive accumulation of Cr^3+^ is dangerous to humans and ultimately causes several severe health problems [18,19]. Moreover, the Cr^3+^ ion is widely utilized in many different industries, including pigment manufacture, leather tanning, wood treatment, and dyeing. It is also one of the metals that causes type 2 diabetes [20,21]. Among existing HMIs, the Cu^2+^ ion is a well-known contaminant in recent environment pollutants which usually accumulate from the release of agricultural and industrial wastes [22]. The detection of HMIs in the environment demands a process which should be specific and less time-consuming. The electrochemical detection method is sensitive and convenient [23], and the results from voltametric and potentiometric ion selection are satisfactory [24,25,26].

Nanostructures are widely employed as electrode materials and exhibit promising sensing performance [27,28,29]. Among different electrode materials, oxide materials with superior adsorption capacity have contributed to notable advancements in the high-performance detection of hazardous species, usually due to their large specific surface area, numerous surface-active sites, and high catalytic efficiency [30]. Metal oxides, including WO_3_, TiO_2_, ZnO, Fe_2_O_3_, etc. [28,31,32,33,34], have been vastly employed as electrodes, but TiO_2_ is primarily beneficial because of its high activity, chemical stability, affordability, and extended reusability [35]. This semiconductor shows excellent optoelectronic and catalytic properties [32]. In this work, we have investigated a novel approach toward a fabricated TiO_2_ NR electrode as a sensor for the detection of three HMIs, i.e., Cr^3+^, Cu^2+^, and Hg^2+^. The manufactured sensor exhibits a high degree of agreement and a promising reproducibility of sensing performance.

## 2. Materials and Methods

### 2.1. Preparation of TiO_2_ NRs

TiO_2_ nanopowder (P25, Degussa, Evonik, Essen, Germany) was exploited as a precursor, and 0.5 g of TiO_2_ was mixed in 10 M NaOH (Sigma-Aldrich, St. Louis, MO, USA) aqueous solution under vigorous stirring. Afterward, the mixture was transferred into a Teflon beaker, and then ITO glass was horizontally placed in the beaker, which was sealed into a stainless-steel autoclave for hydrothermal treatment at 130 °C for 24 h. Once the reaction was finished, the autoclave was left to cool at ambient temperature. The grown thin TiO_2_ films were treated with diluted HCl solution and then washed properly by DI water. Ultimately, TiO_2_ NR films were obtained after a drying process in an oven at ~60 °C.

### 2.2. Characterization of TiO_2_ NRs

Morphological images of TiO_2_ NRs were studied by FESEM (field-emission scanning electron microscopy, Hitachi S-4700, Tokyo, Japan) and TEM (transmission electron spectroscopy, H-7650, Hitachi, Tokyo, Japan). The crystalline structures of TiO_2_ NRs were analyzed by X-ray powder diffraction (XRD, Rigaku, Cu Kα, λ = 1.54178 Å, Tokyo, Japan) by applying a Bragg angle range of 10–80°. The optical characteristics were studied by the UV-vis absorbance (2550 Shimadzu, Kyoto, Japan). Raman spectra (Raman microscope, Renishaw, in 200–1400 cm^−1^ range, Gloucestershire, UK) and FTIR (Nicolet, IR300, Glendale, WI, USA) were subjected to analysis for structural characterizations. Through the utilization of the KRATOS AXIS-Nova instrument (Kyoto, Japan), XPS research was performed to examine the elemental states and surface composition of TiO_2_ NRs. Using TiO_2_ NRs as the electrode and a broad range of HMI concentrations from 10 mM to 200 mM, cyclic voltammetry (C-V) measurements were carried out in 0.1 M phosphate buffer solution (10 mL PBS) using a potentiostat.

## 3. Results and Discussion

### 3.1. Morphological Properties of TiO_2_ NRs

**Figure 1:**
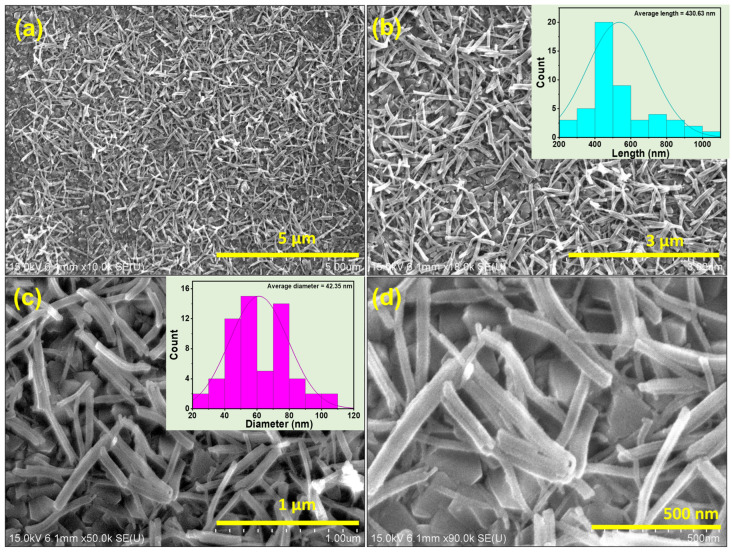
FESEM images of TiO_2_ NR thin film at low (**a**,**b**) and high (**c**,**d**) resolution. Insets show histograms of the length and diameter of TiO_2_ NRs.

To characterize the structural details and surface morphology of the grown TiO_2_ thin film, FESEM studies were performed. At the mode of low magnification, the FESEM image (Figure 1a,b) shows highly dense TiO_2_ NRs covering almost the entire substrate’s surface, and NRs are randomly distributed over ITO glass. The high-resolution images, as shown in Figure 1c,d, reveal an elongated rod-like shape of TiO_2_ with an average length and diameter of ~430.63 nm and ~42.35 nm, respectively, as shown in histograms extracted from FESEM images. Herein, the FESEM images exhibit uniform nanorod (NR) morphology without exhibiting any secondary features like nanoparticles over the surface of NRs.

**Figure 2:**
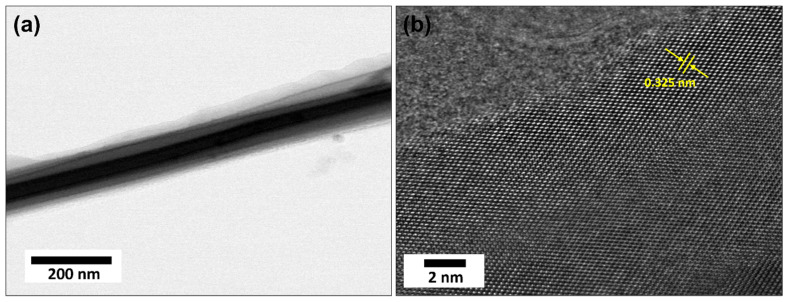
Low-resolution TEM (**a**) and HRTEM (**b**) images of TiO_2_ NR thin film.

Transmission electron microscopy (TEM) provides essential characterization insight into the internal structure, crystallinity, and defects of the nanomaterials. Figure 2a shows a low-resolution TEM image, exhibiting the general morphology of the TiO_2_ NRs with a diameter of ~40 nm (also confirmed by FESEM). In the HRTEM image in Figure 2b, there is a noticeable lattice spacing of about 0.325 nm, which corresponds to the typical lattice spacing of the (101) plane in the TiO_2_ anatase phase [36]. Herein, well-defined diffraction patterns and lattice fringes indicate a high crystallinity of synthesized TiO_2_ NRs.

### 3.2. Crystalline, Optical, and Structural Properties of TiO_2_ NRs

**Figure 3:**
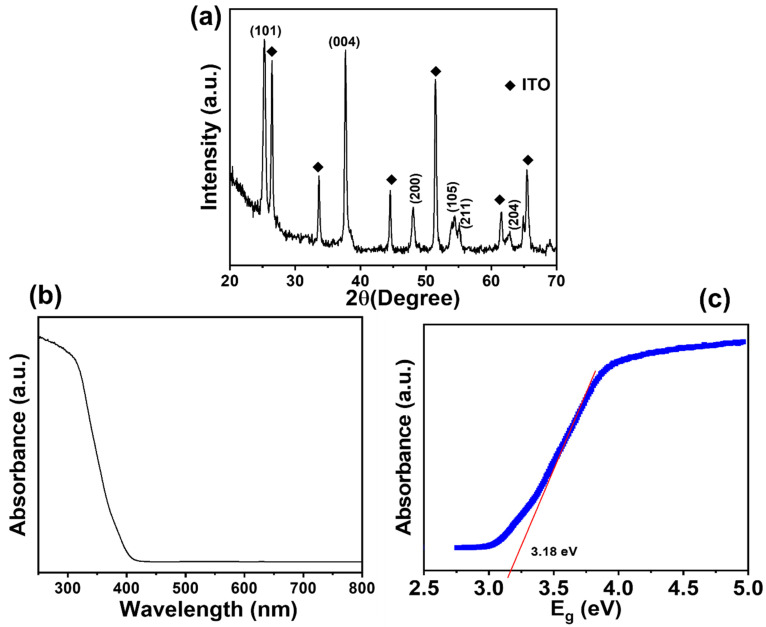
XRD (**a**), UV-vis absorption spectrum (**b**), and its corresponding Tauc plot (**c**) of TiO_2_ NR thin film.

The XRD tool is a widely used technique to characterize the crystallographic properties of nanomaterials, as shown in Figure 3a. The pattern of synthesized TiO_2_ NRs shows the appearance of diffraction peaks at ~25.3°, ~37.8°, ~48.0°, ~54.4°,~55.1°, ~62.8°, and ~68.9°, corresponding to the crystal planes of (101), (004), (200), (105), (211), (204), and (116), respectively [37,38,39]. According to the JCPDS (96-152-6932), these peaks represent the typical anatase phase of TiO_2_. The XRD of the sample reveals a notable (101) peak at ~2θ = 25°, verifying the existence of the typical tetragonal structure of anatase TiO_2_ NRs. Herein, the high intensity of diffraction peak (002) illustrates the crystallinity of TiO_2_ NRs. Additionally, other diffraction peaks are identified at ~26.4°, ~33.7°, ~44.6°, ~51.5°, ~61.4°, and ~65.4°, which are associated with the ITO substrate. Thus, the grown TiO_2_ NRs exhibit a pure phase, as indicated by the characteristic peaks of a single crystal structure.

XRD results reflect the good crystallinity and high purity of the grown TiO_2_ NRs. The Debye–Scherrer equation is utilized to obtain the average crystallite size (D), as explained below:

D = *kλ*/*β*Cos*θ*

where *k* signifies a constant (0.94), *λ* represents the X-ray wavelength (Cu Kα radiation, 1.5406 Å), and *θ* and *β* denote the Bragg’s angle and full width at half maximum (FWHM) of XRD peaks, respectively. Through consideration of the diffraction peak at 25.3°, the FWHM value and crystallite size are found to be 0.32° and 26.58 nm, respectively. The estimated crystallite size is almost similar to the size observed in microscopic analysis.

In the analysis of the UV-vis absorption spectra, as shown in Figure 3b, TiO_2_ NRs exhibit an absorption edge in the UV region of the spectrum. The appearance of the band at ~370–390 nm confirms the anatase TiO_2_ phase [40]. The band gap is determined by analyzing the absorption spectra using a Tauc plot using (αhν)^2^ and photon energy (hν), where α is the absorption coefficient, as shown in Figure 3c. The synthesized anatase TiO_2_ NRs exhibit a band gap of ~3.18 eV, which matches well with the reported work [41].

**Figure 4:**
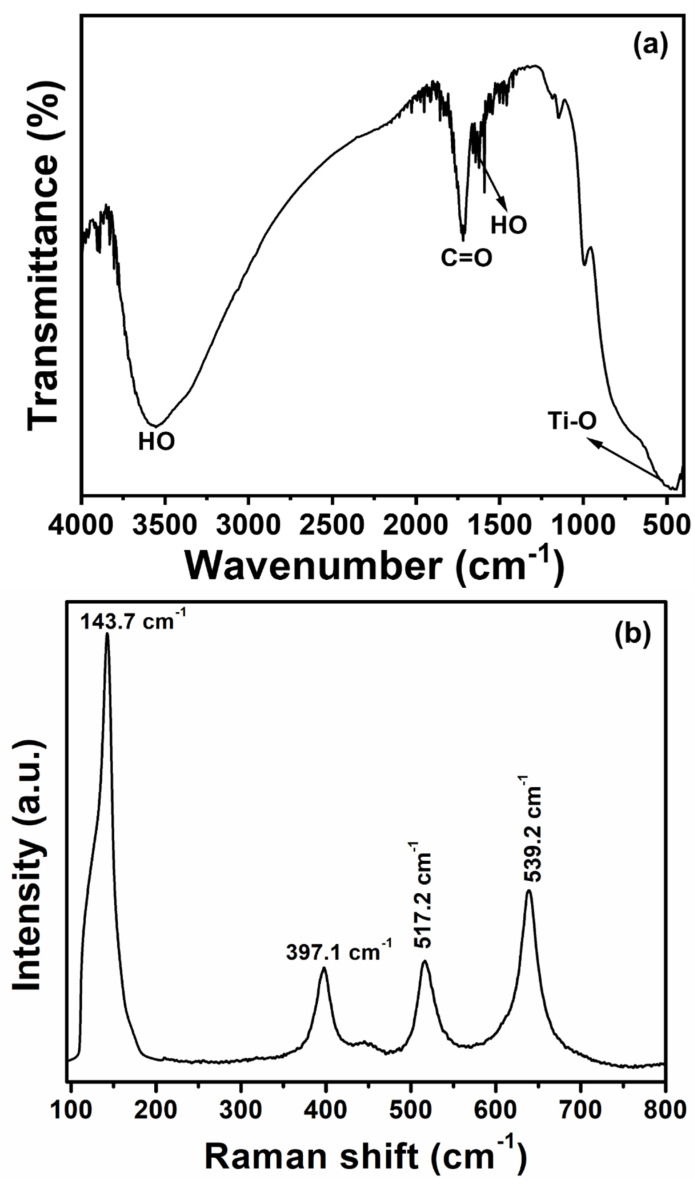
FTIR (**a**) and Raman spectra (**b**) of TiO_2_ NR thin film.

Figure 4a shows FTIR spectroscopy, which is valuable for characterizing the vibrational properties of TiO_2_ NRs, providing insights into their molecular structure, bonding, and surface chemistry. The appearance of peaks around 400–600 cm^−1^ is typically attributed to Ti-O and Ti-O-Ti stretching vibrations, alongside the formation of TiO_2_ NRs [42,43], and is indicative of the crystalline TiO_2_ phase. The broad infrared signal at 3554 cm^−1^ corresponds to O-H stretching vibrations, which show that the TiO_2_ NRs’ surface contains atmospheric hydroxyl groups [44]. A weak IR peak at ~1632 cm^−1^ is associated with the bending vibrations of adsorbed water molecules (H-O-H) [45]. The IR peaks at ~1160 and ~1716 cm^−1^ are ascribed to C-O and C=O species. Thus, the grown TiO_2_ NRs possess a pure TiO_2_ structure with few traces of impurities.

Raman spectroscopy is a crucial tool for the analysis of TiO_2_ NRs, giving valuable insights into their phase composition, crystallinity, and surface characteristics. The Raman spectrum, as shown in Figure 4b, exhibits distinct peaks corresponding to ~143.5 cm^−1^ (Eg) [46], which is normally related to anatase TiO_2_. The other peaks appearing at ~396.7 cm^−1^ (B1g), ~524.1 cm^−1^ (A1g), and ~637.8 cm^−1^ (Eg) further confirm the predominance of anatase in the grown TiO_2_ NRs [47,48]. The peak intensities and positions are significant as they provide insight into the crystallinity of the TiO_2_ NRs. In our work, peaks related to TiO_2_ are sharper and more intense, reflecting the well-formed crystalline structures of TiO_2_ NRs.

### 3.3. XPS Studies of TiO_2_ NRs

**Figure 5:**
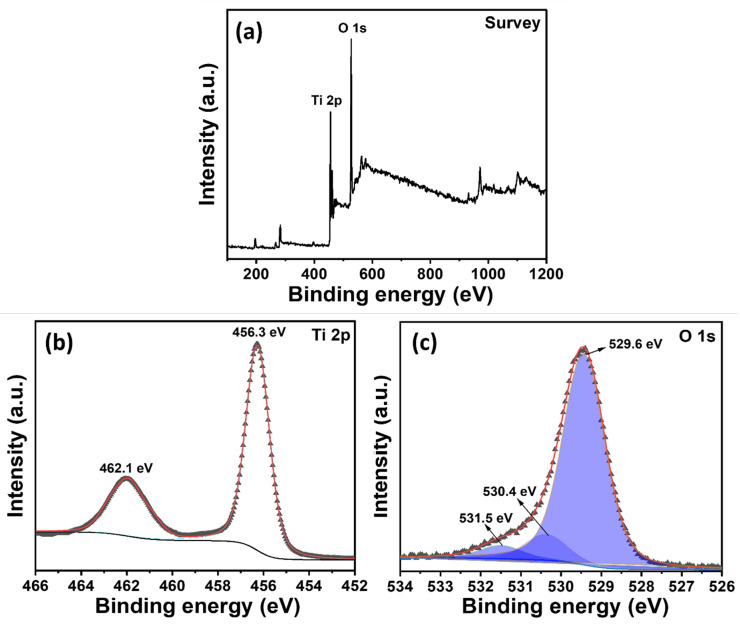
XPS survey (**a**), Ti 2p (**b**), and resolved O 1s (**c**) of TiO_2_ NR thin film.

XPS is a crucial analytical method for examining the elemental makeup and surface chemistry of materials. Figure 5 provides detailed information about the oxidation states of Ti, the bonding environment of O, and the presence of surface species, offering critical insights into the material’s chemical and electronic structure. Figure 5a shows the XPS survey spectra, revealing the presence of titanium (Ti 2p) and oxygen (O 1s) as the primary elements in TiO_2_. In Figure 5b, the Ti 2p peak appears as a doublet binding energy with Ti 2p_3_/_2_ and Ti 2p_1_/_2_, indicating that TiO_2_ is predominantly in the +4 oxidation state [49]. The Ti 2p binding energies are observed at ~456.3 eV (Ti 2p_3_/_2_) and ~462.1 eV (Ti 2p_1_/_2_), which are consistent with the Ti^4^^+^ oxidation state that is characteristic of TiO_2_ [50]. Information about Ti 2p is crucial for confirming the phase purity and oxidation state of Ti in TiO_2_ nanomaterials. Figure 5c exhibits an O 1s peak observed at the central binding energy at ~529.6 eV, which is typically associated with O^2−^ ions in the TiO_2_ lattice [51,52]. The other two binding energies at ~530.4 and ~531.5 eV originated from atmospheric oxygen or moisture adsorbed over TiO_2_ NRs. Therefore, the presence of O^2−^ ions along with the Ti^4^^+^ ion further confirms the formation of TiO_2_ during the hydrothermal process. Moreover, these findings are significant to guiding the further optimization of TiO_2_ NRs in advanced sensor applications.

### 3.4. Selectivity and Sensing Performance of TiO_2_ NR-Modified HMI Sensor

**Figure 6:**
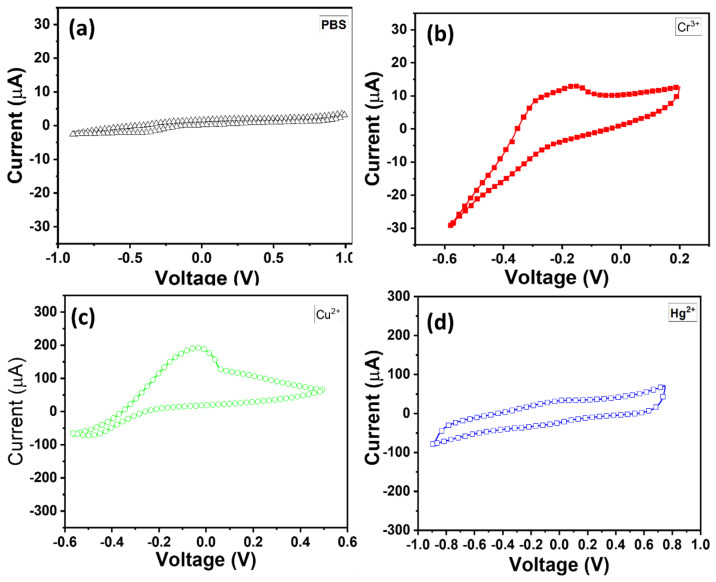
CV measurements of TiO_2_ NR electrode (**a**) in pristine phosphate buffer solution (PBS, pH = 7.0) electrolyte (**a**), and in 10 mM of Cr^3+^ (**b**), Cu^2+^ (**c**), and Hg^2+^ (**d**) in PBS electrolyte.

The detection of HMIs such as Cr^3+^, Cu^2+^, and Hg^2+^ in water is crucial due to their toxic effects on human health and the environment. Electrochemical methods, particularly cyclic voltammetry (CV), are widely used for the detection of these ions due to their sensitivity, selectivity, and relatively low cost. CV provides valuable information about electrochemical properties, including oxidation and reduction processes. The grown TiO_2_ NR-based electrodes in this work offer a promising platform for this purpose owing to their excellent catalytic properties and chemical stability. An electrolyte solution with 0.1 M phosphate buffer solution (PBS, pH = 7.0) has been used for CV measurements. For the selectivity of the electrode, as displayed in Figure 6, the CV plots exhibit the TiO_2_ NR electrode in pristine PBS and PBS with HMIs. Figure 6a displays a weak CV response in the PBS electrolyte, suggesting the reduced sensing behavior of the TiO_2_ NR electrode. After the addition of 10 mM HMIs like Cr^3+^, Cu^2+^, and Hg^2+^ in PBS (Figure 6b–d), the TiO_2_ NR electrode expresses a quick redox current, indicating the sensing response of TiO_2_ NRs. This clearly explains the response of the grown TiO_2_ NR electrode to HMIs like Cr^3+^, Cu^2+^, and Hg^2+^ ions. As shown in Figure 6b–d, the CV scan is conducted by sweeping the potential within a range that includes the redox potentials of Cr^3+^, Cu^2+^, and Hg^2+^, i.e., from −1 V to +1.0 V. The redox peaks corresponding to Cr^3+^ at −0.17 V, Cu^2+^ at −0.028 V, and Hg^2+^ at +0.042 V are identified. These peaks provide qualitative and quantitative information about the presence of each ion. Among HMIs, the TiO_2_ NR electrode displays the highest cathodic current response for the Cu^2+^ ion with a weak anodic current. This suggests that the TiO_2_ NR electrode reflects promising sensing behavior toward Cu^2+^ ions. In the case of Hg^2+^ detection (Figure 6d), during the initial step, the adsorbed Hg^2+^ ions are electrochemically reduced to their metallic forms; for example, Hg^2+^ is reduced to Hg^0^ under an applied negative potential. As for the oxidation process, an oxidation reaction occurs due to a subsequent positive potential applied to reoxidize the reduced metal ions (e.g., Hg^0^ → Hg^2^^+^). This oxidation process produces a characteristic current signal, with the peak position corresponding to the specific metal ion, and the peak intensity is proportional to its concentration. Overall, a higher oxidation peak, as noticed for Cu^2+^, implies the stronger electrocatalytic behavior of the electrode and a faster electron transfer mechanism in an electrochemical system.

**Figure 7:**
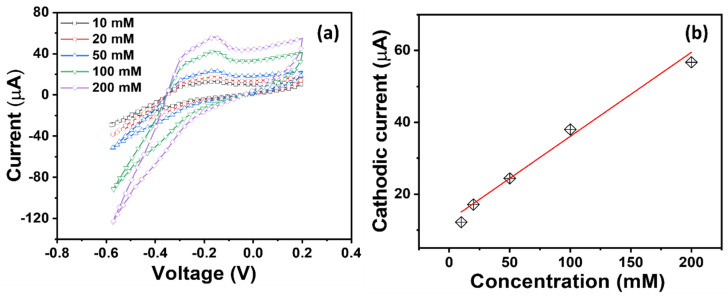
(**a**) CV plots and (**b**) their corresponding calibrated plot of TiO_2_ NR electrode in PBS electrolyte with Cr^3+^ in varied concentrations from 10 to 200 mM.

**Figure 8:**
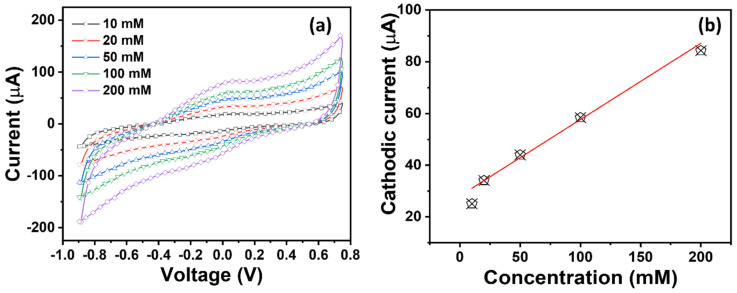
(**a**) CV plots and (**b**) their corresponding calibrated plot of TiO_2_ NR electrode in PBS electrolyte with Hg^2+^ in varied concentrations from 10 to 200 mM.

**Figure 9:**
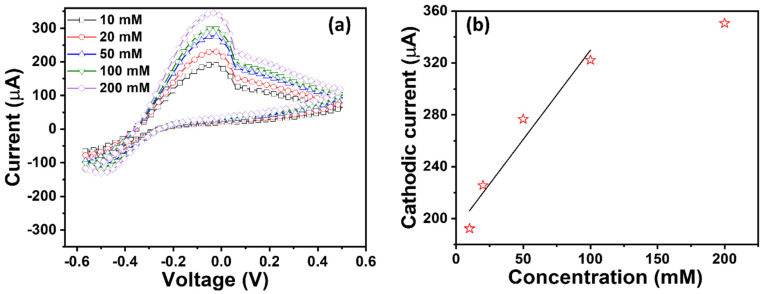
(**a**) CV plots and (**b**) their corresponding calibrated plot of TiO_2_ NR electrode in PBS electrolyte with Cu^2+^ in varied concentrations from 10 to 200 mM.

Figure 7, Figure 8 and Figure 9 display a series of CV plots in PBS electrolytes with different HMIs in their varied concentrations from 10 to 200 mM. Figure 7a shows the variation in the CV plot for different Cr^3+^ ions, depicting an increase in the cathodic current with the increase in Cr^3+^ concentration. In support of this investigation, a calibration curve is depicted by plotting the peak current versus concentration for each of the three HMIs. During linear regression, we aim to fit a straight line to a set of data points. The following linear regression equation is employed to obtain the slope and other parameters.

*y* = *a* + *bx*

where *y* is the dependent variable (output or response), *x* is the independent variable (input or predictor), b is the slope of the line, and a is the y-intercept. To estimate the sensitivity, the slope of the plot is divided by an active area of the electrode (0.015 cm^2^). CV scans with varying concentrations (10–200 mM) of Cr^3+^ are shown in Figure 7a. The current increases linearly as the concentration of Cr^3+^ ions increases. In evaluating the calibration, it is apparent that the TiO_2_ NR electrode exhibits a moderate sensitivity of ~15.6 µAmM^−1^cm^−2^ with a promising dynamic linearity of 10–200 mM, LOD of ~21.7 mM, and R^2^ of 0.096141. Similarly, CV plots of the TiO_2_ NR electrode for Hg^2+^ ions are shown in Figure 8. The redox current response for Hg^2+^ ions is higher than the current response for Cr^3+^ ions. In the analysis of the corresponding calibration plot (Figure 8b), a slightly high sensitivity of ~19.67 µAmM^−1^cm^−2^ with R^2^ = 0.94634 is recorded for Hg^2+^ ions, and a good linear dynamic and LOD are demonstrated.

Additionally, for Cu^2+^ ions, the CV results of TiO_2_ NRs express the highest electrochemical response, as shown in Figure 9. A prominent cathodic peak is seen in Figure 9a, affirming the oxidative behavior of the TiO_2_ NR electrode toward Cu^2+^ ions compared to other ions. The cathodic current dramatically elevates with the increment in Cu^2+^ ion concentration. A higher HMI adsorption at the maximum concentration (200 mM) is made possible by the increase in current, which is correlated with more active sites on the TiO_2_ NR electrode. Through the use of the calibration plot (Figure 9b), improved sensing parameters, like a sensitivity of ~92.2 µAmM^−1^cm^−2^, linear dynamic of 10–100 mM, LOD of ~37 mM, and regression coefficient (R^2^) of ~0.94634, are estimated for Cu^2+^. The sensing parameters are comparable to similarly reported works, as summarized in Table 1. The sensing mechanism of TiO_2_ NR-based electrodes for the electrochemical detection of HMIs is related to their unique structural, surface, and chemical properties. At atmospheric conditions, TiO_2_ NRs contain many oxygenated species on the surface such as hydroxyl groups, which efficiently adsorb HMIs like Cr^3+^, Cu^2+^, and Hg^2+^ in the aqueous solution. Thereafter, the adsorption occurs through binding via electrostatic interactions, coordination bonding, or complex formation between the self-functionalized surfaces of the TiO_2_ electrode and metal ions [53]. This adsorption step enhances the local concentration of HMIs on the electrode, making detection more effective.

**Figure 10:**
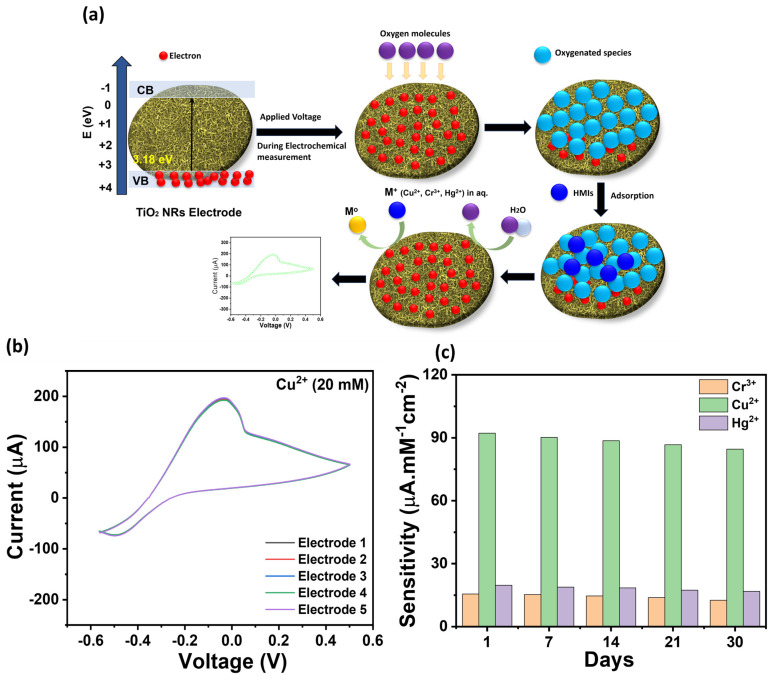
(**a**) An illustration of a possible sensing mechanism of the TiO_2_ NRs electrode for HMIs, (**b**) a reliability test of the TiO_2_ NR-modified HMI sensor (for Cr^3+^, Cu^2+^, and Hg^2+^), and (**c**) histograms exhibiting the sensitivity of the TiO_2_ NR electrode versus days.

A possible sensing mechanism is illustrated in Figure 10a. In general, the band structure of TiO_2_ semiconductors, especially the conduction band, is crucial for the adsorption and oxidation/reduction of the metal ions. When a voltage is applied, firstly, atmospheric oxygenated species are adsorbed on the TiO_2_ NR electrode, and later, electrons are trapped in the conduction band, generating molecular oxygen ions O^2−^. The generated O^2−^ over the TiO_2_ NR electrode can react with HMIs (Cr^3+^, Cu^2+^, and Hg^2+^) and create a layer of metal. Afterward, at high voltage, the conduction band of TiO_2_ NRs releases the electrons during the electrochemical redox process, resulting in an increasing electrical conductivity and current in the presence of HMIs [54,55]. The TiO_2_ NR electrode is highly suitable for Cu^2+^ ions because its electrocatalytic surface provides many active sites for Cu^2+^ ion adsorption. Therefore, as compared to other HMIs, the sensing performance of the TiO_2_ NR electrode for Cu^2+^ ions is superior, showing the highest redox current and excellent sensitivity.

**Table 1:** Sensing performances of the TiO_2_ NR electrode and extracted results from the reported literature.

Electrodes	Target Metals	Dynamic Range	LOD	Sensitivity	Ref.
TiON/TiO_2_	Pb^2+^	10^−5^ to 10^−1^ M	10^−5^ M	-	[56]
Zr/ZrO_2_ nanotube	Cu^2+^	0.05–2 μM	40 nM	-	[57]
Ti/TiO_2_ nanotubes	Cu^2+^	0.01–1 μM	7 nM	-	[58]
SnO_2_/rG0	Cu^2+^	0.4–1.2 μM	0.1141 nM	5.167 mA μM^−1^	[59]
TiO_2_ NRs	Cu^2+^	10–100 mM	37 mM	92.2 µA.mM^−1^.cm^−2^	This work
TiO_2_ NRs	Hg^2+^	10–200 mM	28.5 mM	19.67 µA.mM^−1^.cm^−2^	This work
TiO_2_ NRs	Cr^3+^	10–200 mM	21.7 mM	15.6 µA.mM^−1^.cm^−2^	This work

### 3.5. Reliability and Stability of TiO_2_ NR-Based Tri-HMI Sensor

The detection of Cu^2+^ ions was tested for five electrodes to show the reliability of the TiO_2_ NR-modified HMI sensor. Figure 10b shows that the TiO_2_ NR electrode presents negligible changes in current responses, which suggests no visible variations in the performance of the fabricated electrode. Thus, this study clearly reflects that the TiO_2_ NR electrode for HMI detection is highly reliable. Additionally, the stability tests were carried out by monitoring the performance of manufactured sensors for 30 days in a row. Figure 10c expresses the sensitivity histograms of the TiO_2_ NR electrode versus days. As compared to initial sensitivities, minor variation is detected in the sensitivities of the TiO_2_ NR electrode-based sensors for Cr^3+^, Cu^2+^, and Hg^2+^ ions. In case of Cu^2+^ ions, a slight decrease of 8% in 30 days is noted, but the overall stability is strong. Thus, the fabricated TiO_2_ NR electrode for HMIs (Cr^3+^, Cu^2+^, and Hg^2+^) presents the rapid detection of HMIs with high reliability, sensitivity, and a low LOD.

## 4. Conclusions

In summary, the TiO_2_ NR electrode was successfully constructed and employed for detecting HMIs (Cr^3+^, Cu^2+^, and Hg^2+^). Owing to its distinct structural features and encouraging electrochemical performance, the TiO_2_ NR electrode-based sensor shows a linear relationship between current and the trace concentrations of Cr^3+^, Cu^2+^, and Hg^2+^ ions. The TiO_2_ NR electrode for Cu^2+^ ion detection attains the highest sensitivity of ~92.2 µAmM^−1^cm^−2^, a good linear dynamic of 10–100 mM, an LOD of ~ 37 mM and a regression coefficient (R^2^) of ~0.94634. Thus, this simple, dependable, sensitive, and cost-effective HMI sensor utilizing TiO_2_ NRs shows great potential for environmental monitoring applications. Additional advancements in morphology and composite nanomaterials for electrode materials may be promising in increasing cost-effectiveness, sensitivity, sensing responsiveness, and selectivity.

## Data Availability

The original contributions presented in the study are included in the article, further inquiries can be directed to the author.
